# Laparoscopic procedure is associated with lower morbidity for simultaneous resection of colorectal cancer and liver metastases: *an updated meta-analysis*

**DOI:** 10.1186/s12957-020-02018-z

**Published:** 2020-09-21

**Authors:** Long Pan, Chenhao Tong, Siyuan Fu, Jing Fang, Qiuxia Gu, Shufeng Wang, Zhiyu Jiang, Sarun Juengpanich, Xiujun Cai

**Affiliations:** 1 Key Laboratory of Laparoscopic Technique Research of Zhejiang Province, Department of General Surgery, Sir Run Shaw Hospital, Zhejiang University School of Medicine, Hangzhou, 310016 China; 2 Zhejiang Province Medical Research Center of Minimally Invasive Diagnosis and Treatment of Abdominal Diseases, Hangzhou, 310016 China; 3 Institute of Minimally Invasive Surgery of Zhejiang University, Hangzhou, 310016 China; 4 Department of General Surgery, Shaoxing People’s Hospital, Zhejiang University School of Medicine, Shaoxing, 312000 China; 5 The Third Clinical Medical College, Zhejiang University School of Medicine, Hangzhou, 310016 China

**Keywords:** Colorectal cancer, Synchronous liver metastasis, Laparoscopy, Meta-analysis

## Abstract

**Background:**

It has been demonstrated that simultaneous resection of both primary colorectal lesion and metastatic hepatic lesion is a safe approach with low mortality and postoperative complication rates. However, there are some controversies over which kind of surgical approach is better. The aim of study was to compare the efficacy and safety of laparoscopic surgeries and open surgeries for simultaneous resection of colorectal cancer (CRC) and synchronous colorectal liver metastasis (SCRLM).

**Methods:**

A systemic search of online database including PubMed, Web of Science, Cochrane Library, and Embase was performed until June 5, 2019. Intraoperative complications, postoperative complications, and long-term outcomes were synthesized by using STATA, version 15.0. Cumulative and single-arm meta-analyses were also conducted.

**Results:**

It contained twelve studies with 616 patients (273 vs 343, laparoscopic surgery group and open surgery group, respectively) and manifested latest surgical results for the treatment of CRC and SCRLM. Among patients who underwent laparoscopic surgeries, they had lower rates of postoperative complications (OR = 0.66, 95% CI: 0.46 to 0.96, *P* = 0.028), less intraoperative blood loss (weight mean difference (WMD) = − 113.31, 95% CI: − 189.03 to − 37.59, *P* = 0.003), less time in the hospital and recovering after surgeries (WMD = − 2.70, 95% CI: − 3.99 to − 1.40, *P* = 0.000; WMD = − 3.20, 95% CI: − 5.06 to − 1.34, *P* = 0.001), but more operating time (WMD = 36.57, 95% CI: 7.80 to 65.35, *P* = 0.013). Additionally, there were no statistical significance between two kinds of surgical approaches in disease-free survival and overall survival. Moreover, cumulative meta-analysis indicated statistical difference in favor of laparoscopic surgery in terms of morbidity was firstly detected in the 12th study in 2018 (OR = 0.66, 95% CI: 0.46 to 0.96, *P* = 0.028) as the 95% CI narrowed.

**Conclusion:**

Compared with open surgeries, laparoscopic surgeries are safer (postoperative complications and intraoperative blood loss) and more effective (length of hospital stay and postoperative stay), and it can be considered as the first option for management of SCRLM in high-volume laparoscopic centers.

**Trial registration:**

CRD42020151176

## Introduction

Colorectal cancer (CRC) is a common and lethal disease. Globally, CRC is the third most common cancer in males and second in females, with 1.8 million new cases and almost 861,000 deaths in 2018 according to the World Health Organization GLOBOCAN database. In the USA, annually, approximately 145,600 new cases of large bowel cancer are diagnosed, of which 101,420 are colon and the remainder are rectal cancers [1, 2]. Although CRC mortality has been progressively declining since 1990, currently, 1.7 to 1.9% per year [1], it is still the third leading cause of cancer death in the USA in women and the second common cause of cancer death in men [3, 4]. More importantly, CRC is an extraordinary progressive cancer, and many patients have metastatic lesions at the time of initial diagnosis. Liver is the primary metastatic site for patients with CRC and 15–20% of those patients presenting with synchronous colorectal liver metastases (SCRLM) [5]. These can be resected in one operation or as staged approach, depending on the complexity of the hepatectomy and colectomy, comorbid diseases, and surgeon expertise [6, 7]. The classic approach to SCRLM is to resect the primary lesion, followed by chemotherapy, and subsequent hepatic resection is offered. However, recent studies have demonstrated that simultaneous resection of both primary colorectal lesion and metastatic hepatic lesion is a safe approach with low mortality and postoperative complication rates [8, 9]. Moreover, there are two kinds of surgical approaches to complete simultaneous resection, laparoscopic approach and open approach, respectively [10]. Despite the fact that some studies indicated laparoscopic surgeries had less operating time, intraoperative blood loss, and postoperative pain, most of the studies fail to demonstrate the superiority of laparoscopic surgeries [11]. Moreover, in that case, the outcomes of laparoscopic surgeries associated with current technology and proficiency of surgeons, we believe, will be different from previous studies after several years. Therefore, this meta-analysis was conducted to compare those two kinds of surgical approaches regarding intraoperative and postoperative complications and long-term outcomes on account of the current available literature.

## Methods

### Literature search and selection

This study was conducted according to the Preferred Reporting Items for Systematic Reviews and Meta-Analyses (PRISMA) statement [12]. The protocol for the meta-analysis is registered at PROSPERO (CRD42020151176).

We performed a systemic search of online database including PubMed, Web of Science, Cochrane Library, and Embase to identify relevant studies about comparing open surgeries with laparoscopic surgeries for simultaneous resections of CRC and SCRLM until June 5, 2019. The search strategy was performed using the following terms: colorectal cancer, colorectal cancer liver metastases, synchronous, simultaneous, minimally invasive, laparoscopy, hepatectomy, laparotomy, and open. The detailed search strategy is shown in Additional file 1: Table S1. The references associated with those relevant reviews and meta-analyses were also searched to identify possible additional studies.

The inclusion criteria were as follows: study population (patients with proven or suspected SCRLM (liver metastasis was detected at the same time as detection of CRC)), intervention (laparoscopic vs open surgeries for simultaneous resections), study design (randomized controlled studies or observational studies including cohort and case–control studies), outcome measuring (studies reported at least 1 outcome of the perioperative results or long-term outcomes), the study population which included more than 20 patients (smaller studies were excluded for poor credibility), and studies published as full-length articles.

Some studies were excluded based on the following criteria: abstracts from conferences, case reports, non-comparative studies, review articles, and meta-analyses, and commentary articles were excluded. The study did not clearly distinguish between synchronous liver metastases and metachronous liver metastases. The study failed to distinguish between synchronous resection and staged resection, i.e., hepatectomy only.

### Data extraction and quality assessment

Data were evaluated and extracted by 2 investigators independently (LP and CHT). All the important information were recorded in a Microsoft excel database, such as baseline details, postoperative complications, intraoperative complications (blood loss and operating time), and long-term outcomes (mortality, overall survival rate, and length of hospital stay and postoperative stay). In terms of postoperative complications, they are graded on the Clavien-Dindo Classification, and grade ≥ 3 represents severe complications requiring surgical intervention, the use of organ support, and fatality [13].

Regarding quality assessment of included studies, the Newcastle–Ottawa scale (NOS) were used to evaluate the quality of those twelve studies [14]. The results of quality assessment of included studies are displayed in Additional file 1: Table S2. Disagreements were solved by mutual consensus.

### Statistical analysis

For dichotomous outcomes and continuous outcomes, we used the odds ratio (OR) with 95% confidence intervals (95% CIs) and weight mean difference (WMD) or standardized mean difference (SMD) with 95% CIs to evaluate, respectively. Single-arm meta-analyses and cumulative meta-analyses were conducted for evaluating the postoperative complications between laparoscopic surgery and open surgery groups. Heterogeneities among studies were tested using Cochran Chi-square test and *I*^2^, in which *I*^2^ ≥ 50% suggested significant heterogeneity. A random effects model was used to pool the results when *I*^2^ ≥ 50%, while a fixed effects model was used when low heterogeneity (*I*^2^ < 50%). We also used the Funnel plots, Harbord tests, Peters tests, and Egger tests to detect any publication bias. Harbord tests and Peters tests were used to evaluate the binary data; on the other hand, enumeration variables were processed by Egger tests. *P* < 0.05 was considered as statistical significance (2-sided). All the statistical analyses were conducted by using STATA, version 15.0 (Stata Corporation, College Station, TX).

## Results

### Study selection and quality assessment

**Fig. 1:**
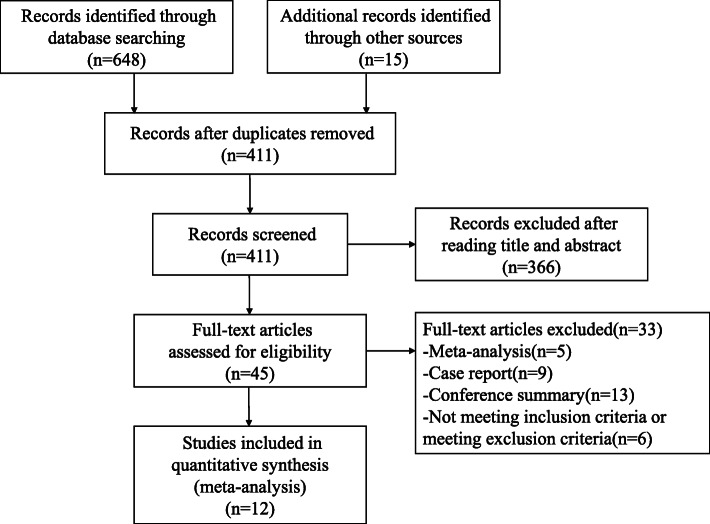
A flow diagram of the inclusion criteria of studies eligible for meta-analysis

Based on the previous search strategy, there were 648 studies after searching from the online databases. Moreover, searching the reference lists and relevant reviews also included fifteen additional publications. Four hundred eleven records remained in all after eliminating duplicates. Next, by means of reading the titles and abstracts, we excluded 366 studies, and there were 45 records to read thoroughly to evaluate the eligibility. Among those 45 records, 33 articles were excluded due to several reasons. The specific reasons of why 33 articles were excluded are displayed in Additional file 1: Table S3. Eventually, we included 12 studies into this meta-analysis [15–26] (Fig. 1). The baseline characteristics and quality evaluation of the included studies are summarized in Tables 1 and 2.

**Table 1:** Demographics and quality assessments of included studies

Study, year	Country	Study period	Study design	Criteria		Matched factors^b^	No. of participants (MH, %)		Age, Mean (SD), y		Female, No. (%)		Quality score
				Inclusion	Exclusion^a^								
							Lap	Open	Lap	Open	Lap	Open	
Ma et al. [23], 2018	China	2014–2017	PSM (R, S)	Primary CRC and SCRLM	1–3	1–3, 5, 7, 11, 12, 15–17, 21, 22,24–26	12 (25)	12 (33)	3^c^ (25)	1^c^ (8)	3 (25)	4 (33)	8
Ivanecz et al [20], .2018	Slovenia	2000–2016	PSM (P, S)	Primary CRC and SCRLM	4–6	1–3, 5, 7, 10–12, 15, 20–27	10 (0)	10 (0)	62 (8)	65 (8)	4 (40)	4 (40)	8
Xu et al. [26], 2017	China	2009–2014	PSM (R, S)	Primary CRC and SCRLM	5, 7	1–3, 5–7, 9, 10, 12, 15–17, 20–22,24– 26, 28	20 (25)	20 (20)	58 (11)	60 (11)	7 (35)	6 (30)	8
Chen et al [16], .2018	Taiwan, China	2009–2017	NM (R, S)	Primary CRC and SCRLM	2, 6, 8–11	1, 2, 7, 11–13, 15, 17, 20, 21, 24, 25, 28, 29	16 (19)	22 (14)	66 (10)	65 (13)	6 (38)	13 (59)	6
Gorgun et al [17], .2017	USA	2006–2015	NM (P, S)	Primary CRC and SCRLM	NR	1, 2, 3, 7, 9–12, 15, 16, 18, 21, 22,24– 26, 28, 29	14 (14)	29 (14)	56 (3)	58 (3)	8 (57)	13 (45)	6
Ratti et al [24], .2016	Italy	2004–2015	PSM (P, S)	Primary CRC and SCRLM	4, 12, 13	1–3, 5, 10, 12, 16– 18, 20–22, 24–26, 28, 29	25 (24)	50 (20)	60 (11)	61 (10)	11 (44)	23 (46)	8
Tranchart et al [25], .2016	France	1997–2013	PSM (R, M)	Liver lesions ≤ 5 cm with segments II– VI	14	1, 2, 7, 10–12, 14, 16, 20, 21,24–26, 28,29	89 (8)	89 (6)	67 (11)	65 (9)	47 (53)	49 (55)	8
Lin et al [22], .2015	China	2008–2012	PSM (P, S)	Primary CRC and SCRLM	15–18	1–3, 5, 7–16, 20, 21, 24–26, 28	7 (0)	36 (17)	60 (3)	57 (10)	2 (29)	15 (58)	8
Jung et al [21], .2013	Korea	2008–2012	CM (P, S)	Primary CRC and SCRLM	5, 19	1, 2, 7, 9–12, 16, 19– 21, 24, 25, 27, 28	24 (25)	24 (25)	60 (43–75)	60 (37–80)	11 (46)	7 (29)	6
Hu et al. [18], 2012	China	2004–2008	CM (R, M)	Metastasis restricted to the left lobe or a segment in the right lobe with size < 6 cm	6, 20, 21, 22	1, 2, 7, 8, 10, 11, 15, 17–19, 28	13 (15)	13 (15)	54 (10)	53 (11)	3 (23)	4 (31)	8
Huh et al [19], .2011	Korea	2003–2008	CM (P, S)	Primary CRC and SCRLM	NR	1–3, 5, 7, 9–16, 25, 28	20^d^ (NR)	20 (NR)	63 (36– 71)	62 (44–85)	7 (35)	5 (25)	8
Chen et al [15], .2011	China	1999–2005	CM (R, S)	Rectal tumor fit for Dixon’s surgery and the number of liver lesions ≤ 2	21, 23, 24	1–10, 13–16	23 (26)	18 (NR)	55 (10)	53 (9)	5 (22)	4 (22)	8

*CRC* Colorectal cancer, *SCRLM* Synchronous liver metastases, *CM* Case-matched, *PSM* Propensity score matching, *MH* Major hepatectomy (≥ 3 segments), *P* prospectively collected data, *R* retrospectively collected data, *M* multicenters, *S* single center, *NR* not report

^a^Exclusion criteria are defined as follows: (1) loss to follow-up; (2) with other malignant tumors; (3) combined multiple organ resection;(4) two stage approaches (colorectal first or liver first; (5) combined radiofrequency ablation; (6) extrahepatic metastases; (7) the application of mixed approaches, initially unresectable SCRLM; (8) with hepatic cellular cancer; (9) with neuroendocrine tumor; (10) with benign liver tumor; (11) with peritoneum seeding; (12) mixed approach (laparoscopic colorectal resection and open hepatectomy or vice versa); (13) follow-up< 12 months; (14) tumors close to the portal pedicle or hepatic veins; (15) incomplete material; (16) with obstructive colorectal cancer; (17) with cancer perforation; (18) with T4 colorectal cancer; (19) minor procedures performed by colorectal surgeon only(liver biopsy or wedge resection of synchronous liver metastases in the liver edge); (20) liver metastasis located in the right hemiliver at a size of > 6cm or requiring the resection of > 2 segments; (21) with a previous history of abdominal surgery or tuberculous peritonitis; (22) with serious cardiopulmonary insufficiency; (23)the rectal tumor fit for Mile’s surgery; (24) the number of liver lesions ≥ 3

^b^Matched factors means the covariate was matched or the difference of the covariate was not statistically significant between two groups and are defined as follows: (1) age; (2) sex; (3) carcinoembryonic antigen; (4) distance between rectal tumors and anus; (5) tumor differentiation; (6) child class; (7) liver metastases size; (8) location of liver metastases (segments 1, 2 ,3, 4b, 4a, 5 ,6, 7, 8); (9) primary tumor size; (10) location of primary tumor (colon/rectum); (11) body mass index; (12) American Society of Anesthesiologists; (13) lymphovascular invasion; (14) depth of primary tumor invasion; (15) postoperative chemotherapy; (16) distribution of metastases (unilobar/bilobar); (17) primary tumor stage; (18) comorbid disease; (19) previous abdomen surgery; (20) types of colorectal and hepatic surgery; (21) number of liver metastases; (22) preoperative chemotherapy; (23) location of liver metastases (anterolateral/posterosuperior); (24) primary tumor T stage; (25) primary nodal status; (26) neoadjuvant chemotherapy; (27) harvested lymph nodes; (28) the type of hepatoenterectomy; (29) recurrence

^c^The number (%) of patients > 60 years

^d^Seven of the 20 patients underwent a total one-step laparoscopic procedure while 13 patients underwent laparoscopic colorectal resection and open liver resection

**Table 2:** Clinicopathological characteristics of included studies

Study	Intervention	Location of primary tumor (colon/rectum)	Primary tumor size, mean (SD), cm	No. of CRLM, mean (SD)	CRLM location (unilober/bilobar)	CRLM size	R0 rate (liver/colorectum)	Neoadjuvant therapy	Mortality	Conversions
Ma et al. [23]	Lap	7/5	NR	1.6 (0.9)	7/5	3.73 ± 2.91	100/100	3 (25)	0	0
	Open	7/5	NR	3.1 (1.8)	5/2	3.02 ± 1.62	100/100	3 (25)	0	
Ivanecz et al. [20]	Lap	4/6	NR	1.4 (0.9)	9/1	2.0 ± 1.2	100/100	7 (70)	0	0
	Open	6/4	NR	1.4 (0.9)	9/1	2.9 ± 1.5	100/100	3 (30)	0	
Xu et al. [26]	Lap	15/5	3.2 (1.0)	6^a^	18/2	2.99 ± 1.55	100/100	6 (30)	0	0
	Open	15/5	3.8 (1.2)	6	17/3	3.19 ± 1.53	100/100	4 (20)	0	
Chen et al [16].	Lap	NR/NR	4.0 (2.0)	4^a^	NR	5.5 ± 4.2	NR/NR	NR	0	1
	Open	NR/NR	5.0 (3.0)	3	NR	4.7 ± 3.7	NR/NR	NR	0	
Gorgun et al. [17]	Lap	6/8	3.7 (0.7)	1.6 (0.3)	12/2	2.4 ± 0.7	86/NR	6 (43)	0	0
	Open	14/15	3.7 (0.5)	2.1 ± 0.2	19/10	2.7 ± 0.2	93/NR	19 (66)	1	
Ratti et al [24].	Lap	13/12	NR	2.40 ± 1.27	13/12	3.65 ± 2.67	96/100	20 (80)	0	1
	Open	27/23	NR	2.35 ± 1.34	27/23	3.94 ± 2.47	98/98	39 (78)	0	
Tranchart et al. [25]	Lap	48/41	NR	1.4 ± 0.6	78/11	2.9 ± 1.9	83/NR	11 (12)	2	6
	Open	51/38	NR	1.5 ± 0.7	81/8	2.8 ± 2.0	90/NR	20 (22)	0	
Lin et al [22].	Lap	3/4	5.3 (1.1)	1.9 ± 0.9	NR	3.3 ± 1.8	100/NR	3 (27)	0	0
	Open	19/17	5.7 (1.9)	2.1 ± 1.0	NR	4.2 ± 2.2	100/NR	13 (36)	0	
Jung et al [21].	Lap	18/6	5.23 (2.13)	NR	23/1	2.81 ± 1.72	100/96	NR	0	0
	Open	16/8	5.56 (1.93)	NR	18/6	3.23 ± 2.21	100/100	NR	0	
Hu et al [18].	Lap	8/5	NR	NR	NR	3.2 ± 1.0	NR/NR	0 (0)	0	0
	Open	8/5	NR	NR	NR	3.5 ± 0.9	NR/NR	0 (0)	0	
Huh et al [19].	Lap	7/13	4 (2–10)	2 (1–7)	17/3	2 (0.9–5.5)	100/NR	NR	0	0
	Open	11/9	4.7 (3–7)	2 (1–8)	16/4	2.4 (1–10)	100/NR	NR	0	
Chen et al [15].	Lap	0/23	2.5 (0.9)	NR	NR	5.5 ± 1.2	NR/NR	NR	0	0
	Open	0/18	2.3 (1.0)	NR	NR	5.6 ± 1.4	NR/NR	NR	0	

*Lap* Laparoscopic surgery, *NR* Not report

^a^The number of patients with CRLM ≥ 3

### Intraoperative outcomes

All the included studies reported the intraoperative blood loss and operating time. Patients who received laparoscopic surgeries had less intraoperative blood loss according to the results (WMD = − 113.31, 95% CI: − 189.03 to − 37.59, *P* = 0.003). This meta-analysis also indicates that the operating time surgeons spent on patients who underwent laparoscopic surgeries were much longer (WMD = 36.57, 95% CI: 7.80 to 65.35, *P* = 0.013). Detailed results of intraoperative outcomes were displayed in the Table 3 and Additional Figure S1.

**Table 3:** Secondary outcomes in this meta-analysis

Outcome of interest	No. of studies	WMD/OR	95% CIs	*P* value	*I*^*2*^ (%)
Operative time	12	36.57	7.80 to 65.35	0.013	82.4
Blood loss	12	− 113.31	− 189.03 to − 37.59	0.003	91.4
Hospital stay	7	− 2.70	− 3.99 to − 1.40	< 0.001	53.6
Postoperative stay	4	− 3.20	− 5.06 to − 1.34	0.001	55.2
1-year DFS	4	1.05	0.59 to 1.86	0.86	0
3-year DFS	4	0.66	0.41 to 1.08	0.097	7.5
1-year OS	5	0.56	0.23 to 1.33	0.187	0
3-year OS	6	0.94	0.53 to 1.65	0.822	0
5-year OS	3	0.69	0.29 to 1.68	0.417	0

*DFS* Disease-free survival, *OS* Overall survival

### Postoperative complications

**Fig. 2:**
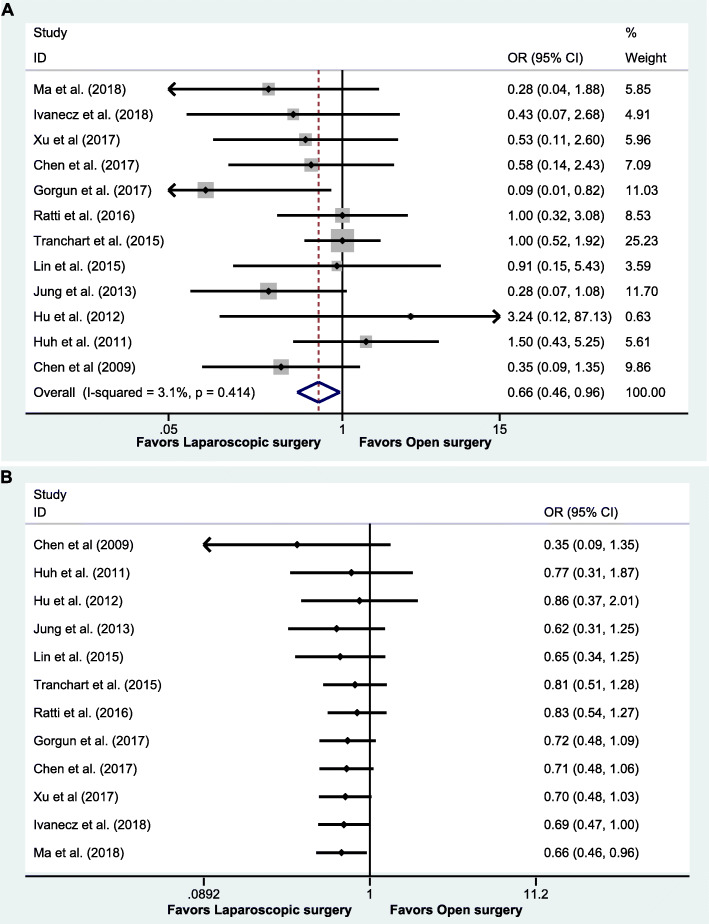
Meta-analysis for primary outcomes. **a** Forest plot of meta-analysis in postoperative complications. **b** Cumulative meta-analysis of postoperative complications

The postoperative complications rate between laparoscopic surgeries and open surgeries was 0.208 (95% CI: 0.161 to 0.254) and 0.325 (95% CI: 0.275 to 0.375), respectively. This meta-analysis suggested that the postoperative complications were significantly lower among patients who underwent laparoscopic surgeries (OR = 0.66, 95% CI: 0.46 to 0.96, *P* = 0.028) (Fig. 2a), without heterogeneity (*I*^2^ = 3.1%, *P* = 0.414). When it comes to the laparoscopic surgery, we assume that the results may be associated with current technology and proficiency of general surgeons. Hence, we did a cumulative meta-analysis, and it indicated statistical difference in favor of the laparoscopic surgery which was firstly detected in the 12th study in 2018 (OR = 0.66, 95% CI: 0.46 to 0.96, *P* = 0.028) as the 95% CI narrowed (Fig. 2b).

**Fig. 3:**
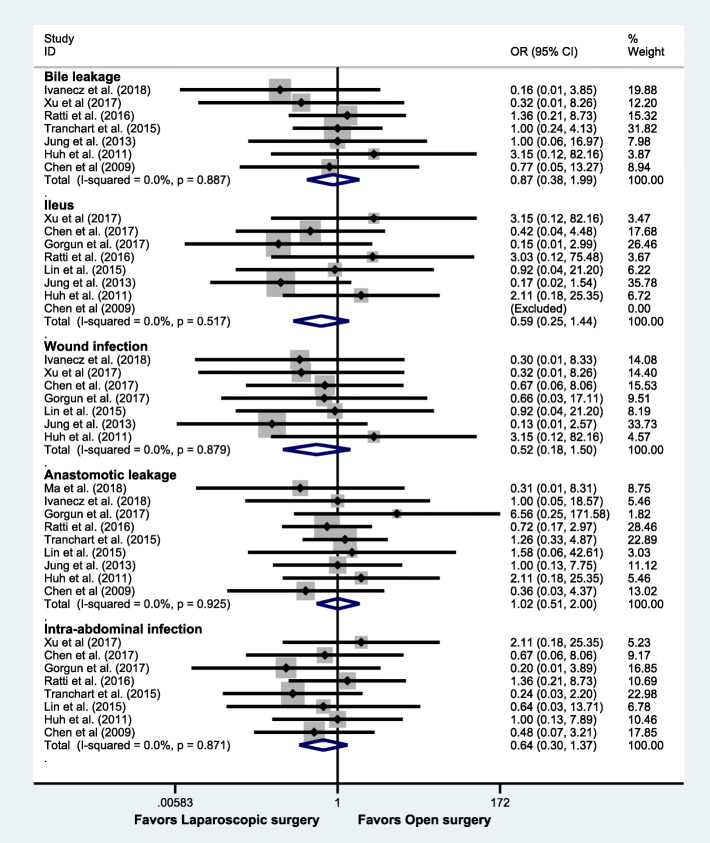
Subgroup analysis based on the types of morbidity

Subsequently, we did subgroup analyses according to the type of morbidity. Although there was no statistical significance in each subgroup (bile leakage: OR = 0.87, 95% CI: 0.38 to 1.99, *P* = 0.74; ileus: OR = 0.59, 95% CI: 0.25 to 1.44, *P* = 0.248; wound infection: OR = 0.52, 95% CI: 0.18 to 1.50, *P* = 0.224; anastomotic leakage: OR = 1.02, 95% CI: 0.51 to 2.00, *P* = 0.965; intra-abdominal infection: OR = 0.64, 95% CI: 0.30 to 1.37, *P* = 0.250), the tendencies in the ileus, wound infection, and intra-abdominal infection were in favor of laparoscopic surgery (Fig. 3).

Additionally, these outcomes were also divided into 2 subgroups based on their study types (Propensity vs Non-propensity). Despite the fact that there was no statistical significance on postoperative complications in the Propensity group (OR = 0.81, 95% CI: 0.51 to 1.30, *P* = 0.388), the result in the Non-propensity group showed that those patients with laparoscopic surgeries were less likely to develop postoperative complications (OR = 0.49, 95% CI: 0.27 to 0.88, *P* = 0.016). However, the outcomes in each subgroup still showed consistency, compared with the overall outcomes. When we subdivided postoperative complications to 2 groups based on the Clavien classification, both grade ≥ III complications and grade < III complications showed no statistical significance between 2 kinds of surgeries (OR = 0.94, 95% CI: 0.52 to 1.71, *P* = 0.835; OR = 0.69, 95% CI: 0.41 to 1.16, *P* = 0.159, respectively). Detailed results of subgroup analyses were displayed in the Additional Figure S1.

### Other outcomes

Based on those twelve studies, this meta-analysis suggested that there was no statistical significance between laparoscopic surgery and open surgery in disease-free survival (1 year: OR = 1.05, 95% CI: 0.59 to 1.86, *P* = 0.86; 3 years: OR = 0.66, 95% CI: 0.41 to 1.08, *P* = 0.097) and overall survival (1 year: OR=0.56, 95% CI: 0.23 to 1.33, *P* = 0.187; 3 years: OR = 0.94, 95% CI: 0.53 to 1.65, *P* = 0.822; 5 years: OR = 0.69, 95% CI: 0.29 to 1.68, *P* = 0.417). Regarding to length of hospital stay and postoperative stay, the results indicated that patients who had laparoscopic surgeries spent less time in the hospital and recovering after surgeries (WMD = − 2.70, 95% CI: − 3.99 to − 1.40, *P* = 0.000; WMD = − 3.20, 95% CI: − 5.06 to − 1.34, *P* = 0.001). Detailed results of other outcomes were displayed in Table 3 and Additional file Figure S1.

### Sensitivity analysis and publication bias

In consideration of the influence of adjuvant chemotherapy on the overall survival and disease-free survival, the administration of postoperative chemotherapy was comparable between the laparoscopic and open groups in nine studies, while the remaining three studies [21, 24, 25] did not report the details about postoperative chemotherapy. Therefore, we did a sensitivity analysis by removing those three studies, and the results were consistent with original outcomes (overall survival: 1 year, 0.54 (95% CI 0.16–1.59; 5 years, 0.66 (95% CI 0.16–2.69); disease-free survival: 1 year, 1.31 (95% CI 0.50–3.41); 3 years, 0.80 (95% CI 0.34–1.86)). Besides, general sensitivity analyses by omitting each study have confirmed this meta-analysis has good stability and strong robustness. Funnel plots of all those results showed a symmetric distribution. Harbord, Peters, and Egger tests were used to demonstrate that there was no publication bias among those studies of primary outcomes. Specifically, the funnel plot of postoperative complications is symmetric and none of the included studies was outside the 95% CI (Harbord test *P* = 0.524; Peter’s test *P* = 0.155). Detailed results of sensitivity analysis and publication bias are shown in Additional file 1: Figure S2 and S3.

## Discussion

This meta-analysis included studies from 2009 to 2018 to compare the efficacy and safety of laparoscopic surgeries and open surgeries in patients with CRC and SCRLM. It includes twelve studies with 616 patients (273 vs 343) and manifests latest surgical results for the treatment of CRC and SCRLM. All included studies were of relatively high quality, and the heterogeneity among primary outcomes was very low. Besides, sensitivity analyses indicated that the results were not affected by any individual study.

 Recently, there is a meta-analysis about this issue published in June 2019 by Ye et al. [27]. Their study showed that the laparoscopic surgery group had less intraoperative blood loss and blood transfusions and quicker postoperative recovery. However, there was no significant difference between the two groups in postoperative complications. Compared with their meta-analysis, our study has a positive result over the rate of overall postoperative complications. We found the fact that this presenting meta-analysis included 3 more studies, Xu et al. [26], Jung et al. [21], and Huh et al.’s [19] and excluded one of their studies, Takasu et al.’s [28], due to the small size of samples (7 vs 7). This meta-analysis indicated that postoperative complications were significantly less among patients who underwent laparoscopic surgeries (*P* = 0.028). Since the fact that laparoscopic surgery is a relatively newly developed surgical approach compared with laparotomy, and requires special equipment and skilled general surgeons who must have rich experience, the outcomes can be influenced by the time and the quality of medical care of each included study. Therefore, we conducted a cumulative meta-analysis to confirm our point. It indicated that statistical difference in favor of laparoscopic surgery was firstly detected in the 12th study in 2018 (the latest one) as the 95% CI narrowed. This result illustrated the reason why prior systemic reviews and meta-analyses did not have statistical significance of this variable. Then, in order to further explore the differences in different types of complications between laparoscopic procedure and open procedure, a subgroup meta-analysis was performed. We found that laparoscopic procedure can reduce ileus, wound infection, and intra-abdominal infection compared with open approach to a certain degree, although they did not reach statistical differences, which may provide useful value for clinical practice. We also conducted another subgroup analysis based on their study types (Propensity vs Non-propensity). Even though in the Propensity subgroup, there was no statistical significance (*P* = 0.388), it still showed consistency, compared with the overall outcomes. The result in the Non-propensity group showed that those patients with laparoscopic surgery were less likely to develop postoperative complications (*P* = 0.016). Besides, a randomized controlled trial is ongoing comparing robotic versus open surgery for simultaneous resection of CRC and CRLM (NCT02642978), and the mid-term outcomes (58 vs 57) indicated lower liver-related complication morbidity (10.3% vs 28.1%, *P* = 0.016) was observed in robotic group [29]. Hence, we believe that as more studies are included, the effect sizes will be stable, and 95% CI will become narrower.

We also analyzed the intraoperative outcomes, specifically intraoperative blood loss. As we expected, patients receiving laparoscopic surgeries had less intraoperative blood loss (*P* = 0.003). This result is quite convincing due to the fact that laparoscopic surgery is a less invasive intervention than laparotomy, and it has smaller incisions, less blood transfusions, and fewer chances to cause additional injuries. Subsequently, due to less intraoperative and postoperative complications, when it comes to the length of hospital stay and postoperative stay, patients with laparoscopic surgeries spent less time in the hospital and recovering after surgeries (*P* = 0.000; *P* = 0.001). Another important factor that should be considered is long-term outcomes. This meta-analysis suggested that the results were similar between laparoscopic surgeries and open surgeries in disease-free survival and overall survival. This result also demonstrated that the approaches of surgeries only affected intraoperative and postoperative complications but not long-term outcomes.

Certain limitations presenting in this meta-analysis have an impact on the outcomes. Firstly, none of the included studies were randomized controlled trials and the number of included studies was not large enough, which will both definitely increase selection and procedure bias. Secondly, one of the most important limitations is heterogeneity, and several situations may cause this. There are twelve different studies included and 616 patients with different conditions are treated by different surgeons with various experience. Even though all patients had SCRLM, their oncological conditions, different locations of liver metastasis, and underlying diseases may affect the outcomes. Although the primary outcomes showed low heterogeneity, other outcomes such as intraoperative bleeding, operating time, and postoperative and hospital stay demonstrated high heterogeneities.

## Conclusion

Compared with open surgeries, laparoscopic surgeries are safer (postoperative complications and intraoperative blood loss) and more effective (length of hospital stay and postoperative stay). These findings suggest that laparoscopic surgeries should be considered the first option for the management of SCRLM, especially when confronted with eligible cases.

## Supplementary information

**Additional file 1: Figure S1.** Forest plot of meta-analysis. (A) Blood loss. (B) Operating time. (C) Subgroup analysis of postoperative complications. (D) Clavien grade<III complications. (E) Clavien grade ≥III complications. (F) Hospital stay. (G) Postoperative stay. (H) One-year overall survival rate. (I) Three-year overall survival rate. (J) Five-year overall survival rate. **Figure S2.** Sensitivity of all the outcomes. (A) Blood loss. (B) Operating time. (C) Postoperative complications. (D) Clavien grade<III complications. (E) Clavien grade ≥III complications. (F) Hospital stay. (G) Postoperative stay. (H) One-year overall survival rate. (I) Three-year overall survival rate. (J) Five-year overall survival rate. **Figure S3.** Publication bias of all the outcomes. (A)-(J) Funnel plot of Blood loss, Operating time, Postoperative complications, Clavien grade<III complications, Clavien grade ≥III complications, Hospital stay, Postoperative stay, One-year overall survival rate, Three-year overall survival rate, Five-year overall survival rate. (K) Quantitative assessment for publication bias. **Table S1.** Search Strategy for Each Database. **Table S2.** Quality Assessment of Included Studies. **Table S3.** Excluded Articles and Reasons for Exclusion.

## Data Availability

All data generated or analyzed during this study are included in this published article.

## Abbreviations

CRC: Colorectal carcinoma
SCRLM: Synchronous colorectal liver metastasis
PRISMA: Preferred Reporting Items for Systematic Reviews and Meta-Analyses
OS: Overall survival
ORs: Odds ratios
CI: Confidence intervals
WMD: Weight mean difference
SMD: Standardized mean difference
NOS: Newcastle–Ottawa scale
