# Supplementary material for: Laparoscopic procedure is associated with lower morbidity for simultaneous resection of colorectal cancer and liver metastases: an updated meta-analysis
Source: World J Surg Oncol. 2020 Sep 21;18:251. doi: 10.1186/s12957-020-02018-z (PMC7507629; doi:10.1186/s12957-020-02018-z)
Supplement: Supplementary file 1 — Additional file 1: Figure S1. Forest plot of meta-analysis. (A) Blood loss. (B) Operating time. (C) Subgroup analysis of postoperative complications. (D) Clavien grade<III complications. (E) Clavien grade ≥III complications. (F) Hospital stay. (G) Postoperative stay. (H) One-year overall survival rate. (I) Three-year overall survival rate. (J) Five-year overall survival rate. Figure S2. Sensitivity of all the outcomes. (A) Blood loss. (B) Operating time. (C) Postoperative complications. (D) Clavien grade<III complications. (E) Clavien grade ≥III complications. (F) Hospital stay. (G) Postoperative stay. (H) One-year overall survival rate. (I) Three-year overall survival rate. (J) Five-year overall survival rate. Figure S3. Publication bias of all the outcomes. (A)-(J) Funnel plot of Blood loss, Operating time, Postoperative complications, Clavien grade<III complications, Clavien grade ≥III complications, Hospital stay, Postoperative stay, One-year overall survival rate, Three-year overall survival rate, Five-year overall survival rate. (K) Quantitative assessment for publication bias. Table S1. Search Strategy for Each Database. Table S2. Quality Assessment of Included Studies. Table S3. Excluded Articles and Reasons for Exclusion. [file 12957_2020_2018_MOESM1_ESM.zip › Additional Figures.docx]

|  | | | | Study |  | % |
| --- | --- | --- | --- | --- | --- | --- |
| Study | % | Study | % | ID | OR (95% CI) | Weight |

ID WMD (95% CI)

Weight ID

WMD (95% CI)

Weight

Ma, K., et al. (2018)

-372.00 (-616.61, -127.39) 4.94

Ivanecz, A., et al. (2018) Xu, X., et al (2017)

Chen, Y. W., et al. (2017)

-57.00 (-100.86, -13.14) 9.85

-86.00 (-135.00, -37.00) 9.77

44.00 (-180.09, 268.09) 5.38

Gorgun, E., et al. (2017) Ratti, F., et al. (2016) Tranchart et al. (2015) Lin, Q., et al. (2015)

Jung, K. U., et al. (2013)

-231.00 (-277.46, -184.54) 9.81

-223.00 (-335.56, --110.44) 8.32

41.00 (-22.98, 104.98) 9.50

--110.00 (-215.48, -4.52) 8.51

61.00 (-58.72, 180.72) 8.12

Hu, M. G., et al. (2012)

Huh, J. W., et al. (2011) Chen, K. Y., et al (2009)

-15.00 (-94.42, 64.42) 9.16

-140.00 (-301.46, 21.46) 6.96

-315.00 (-370.51, -259.49) 9.66

Overall (I-squared = 91.4%, p = 0.000)

--113.31 (-189.03, -37.59) 100.00

NOTE: Weights are from random effects analysis

Ma, K., et al. (2018)

Ivanecz, A., et al. (2018) Xu, X., et al (2017)

Chen, Y. W., et al. (2017) Gorgun, E., et al. (2017) Ratti, F., et al. (2016) Tranchart et al. (2015) Lin, Q., et al. (2015)

Jung, K. U., et al. (2013)

Hu, M. G., et al. (2012)

Huh, J. W., et al. (2011) Chen, K. Y., et al (2009)

Overall (I-squared = 82.4%, p = 0.000)

54.00 (-70.17, 178.17) 3.64

4.00 (-67.04, 75.04) 6.70

-1.00 (-49.97, 47.97) 8.53

93.00 (28.04, 157.96) 7.18

-20.00 (-40.80, 0.80) 10.69

111.00 (51.06, 170.94) 7.59

24.00 (-5.01, 53.01) 10.15

109.00 (63.03, 154.97) 8.78

62.00 (17.47, 106.53) 8.91

-37.00 (-71.60, -2.40) 9.73

87.00 (28.25, 145.75) 7.69

8.00 (-17.42, 33.42) 10.40

36.57 (7.80, 65.35) 100.00

NOTE: Weights are from random effects analysis

-617

Laparoscopic surgery 00

# A

Open Surgery

617

-178 0 178

Laparoscopic surgery Open Surgery

# B

.0109 1 91.7

Propensity

Ma, K., et al. (2018)

Ivanecz, A., et al. (2018) Xu, X., et al (2017) Ratti, F., et al. (2016) Dagher, I., et al. (2015) Lin, Q., et al. (2015)

Subtotal (I-squared = 0.0%, p = 0.779)

.

Non-propensity

Chen, Y. W., et al. (2017) Gorgun, E., et al. (2017) Jung, K. U., et al. (2013)

Hu, M. G., et al. (2012)

Huh, J. W., et al. (2011) Chen, K. Y., et al (2009)

Subtotal (I-squared = 33.6%, p = 0.184)

.

Overall (I-squared = 3.1%, p = 0.414)

0.28 (0.04, 1.88) 5.85

0.43 (0.07, 2.68) 4.91

0.53 (0.11, 2.60) 5.96

1.00 (0.32, 3.08) 8.53

1.00 (0.52, 1.92) 25.23

0.91 (0.15, 5.43) 3.59

0.81 (0.51, 1.30) 54.08

0.58 (0.14, 2.43) 7.09

0.09 (0.01, 0.82) 11.03

0.28 (0.07, 1.08) 11.70

3.24 (0.12, 87.13) 0.63

1.50 (0.43, 5.25) 5.61

0.35 (0.09, 1.35) 9.86

0.49 (0.27, 0.88) 45.92

0.66 (0.46, 0.96) 100.00

Laparoscopic surgery Open Surgery

# C

Study ID

OR (95% CI)

%

Weight

Study ID

OR (95% CI)

%

Weight

Study ID

WMD (95% CI)

%

Weight

Study

ID

WMD (95% CI)

%

Weight

Ma, K., et al. (2018)

-6.00 (-12.62, 0.62) 6.78

Ratti, F., et al. (2016)

-5.00 (-7.04, -2.96) 30.94

Jung, K. U., et al. (2013)

-2.00 (-4.56, 0.56) 25.44

Hu, M. G., et al. (2012)

-2.00 (-3.54, -0.46) 36.84

Xu, X., et al (2017)

(Excluded) 0.00

Overall (I-squared = 55.2%, p = 0.082)

-3.20 (-5.06, -1.34) 100.00

NOTE: Weights are from random effects analysis

-12.6 00

Ma, K., et al. (2018)

0.17 (0.01, 3.90) 10.83

Ivanecz, A., et al. (2018)

-7.00 (-19.03, 5.03) 1.12

Ivanecz, A., et al. (2018)

0.26 (0.02, 3.06) 12.17

Chen, Y. W., et al. (2017)

-1.00 (-4.51, 2.51) 9.67

Xu, X., et al (2017)

0.30 (0.03, 3.15) 12.85

Gorgun, E., et al. (2017)

-4.00 (-4.64, -3.36) 30.90

Chen, Y. W., et al. (2017)

0.42 (0.04, 4.48) 10.67

Tranchart et al. (2015)

-2.00 (-4.80, 0.80) 13.05

Tranchart et al. (2015)

1.60 (0.68, 3.79) 37.48

Lin, Q., et al. (2015)

-1.00 (-3.58, 1.58) 14.36

Jung, K. U., et al. (2013)

1.57 (0.24, 10.37) 7.89

Huh, J. W., et al. (2011)

0.00 (-3.72,, 3.72) 8.88

Huh, J. W., et al. (2011)

1.00 (0.13, 7.89) 8.11

Chen, K. Y., et al (2009)

-4.00 (-5.61, -2.39) 22.02

Hu, M. G., et al. (2012)

(Excluded) 0.00

Overall (I-squared = 53.6%, p = 0.044)

-2.70 (-3.99, -1.40) 100.00

Overall (I-squared = 0.0%, p = 0.504)

0.94 (0.52, 1.71) 100.00

NOTE: Weights are from random effects analysis

-19

0

Laparoscopic surgery Open Surgery

19

Ma, K., et al. (2018)

0.60 (0.08, 4.45) 7.28

Ivanecz, A., et al. (2018)

1.00 (0.11, 8.95) 4.66

Xu, X., et al (2017)

1.00 (0.13, 7.89) 5.24

Chen, Y. W., et al. (2017)

0.78 (0.16, 3.90) 9.96

Tranchart et al. (2015)

0.62 (0.26, 1.48) 38.76

Jung, K. U., et al. (2013)

0.09 (0.01, 0.77) 22.32

Hu, M. G., et al. (2012)

3.24 (0.12, 87.13) 1.30

Huh, J. W., et al. (2011)

1.56 (0.42, 5.76) 10.48

Overall (I-squared = 0.0%, p = 0.522)

0.69 (0.41, 1.16) 100.00

Laparoscopic surgery Open Surgery

12.6

.00988 1 101

Laparoscopic surgery Open Surgery

# D

.00723

1

Laparoscopic surgery

# E

Open Surgery

138

# F G

Study

% Study

% Study %

ID OR (95% CI)

Weight ID

OR (95% CI)

Weight ID

OR (95% CI)

Weight

Jung, K. U., et al. (2013)

0.71 (0.23, 2.23)

58.72

Hu, M. G., et al. (2012)

1.00 (0.19, 5.29)

23.23

Chen, K. Y., et al (2009)

0.23 (0.01, 5.16)

18.05

Overall (I-squared = 0.0%, p = 0.717)

0.69 (0.29, 1.68)

100.00

.0105 1 95.4

Ivanecz, A., et al. (2018)

2.25 (0.17, 29.77) 3.23

Xu, X., et al (2017)

1.50 (0.43, 5.25)

16.17

Chen, Y. W., et al. (2017)

0.33 (0.08, 1.36)

28.08

Hu, M. G., et al. (2012)

1.00 (0.21, 4.67)

13.06

Huh, J. W., et al. (2011)

1.23 (0.35, 4.31)

17.78

Chen, K. Y., et al (2009)

0.83 (0.24, 2.90)

21.69

Overall (I-squared = 0.0%, p = 0.657)

0.94 (0.53, 1.65)

100.00

Ma, K., et al. (2018)

1.00 (0.06, 18.08) 6.51

Chen, Y. W., et al. (2017)

0.12 (0.01, 2.49) 26.35

Tranchart et al. (2015)

0.59 (0.14, 2.53) 34.29

Hu, M. G., et al. (2012)

1.00 (0.06, 17.90) 6.55

Chen, K. Y., et al (2009)

0.74 (0.16, 3.47)

26.31

Ivanecz, A., et al. (2018)

(Excluded)

0.00

Overall (I-squared = 0.0%, p = 0.844)

0.56 (0.23, 1.33)

100.00

.00623

Laparoscopic surgery 1 Open Surgery

# H

161

.0336 1 29.8

# I J

Figure S1. Forest plot of meta-analysis. (A) Blood loss. (B) Operating time. (C) Subgroup analysis of postoperative complications.

(D) Clavien grade<III complications. (E) Clavien grade ≥III complications. (F) Hospital stay. (G) Postoperative stay

1. One-year overall survival rate. (I) Three-year overall survival rate. (J) Five-year overall survival rate


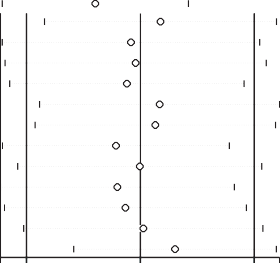
Ma, K., et al. (2018)

Ivanecz, A., et al. (2018) Xu, X., et al (2017)

Chen, Y. W., et al. (2017) Gorgun, E., et al. (2017) Ratti, F., et al. (2016) Tranchart et al. (2015) Lin, Q., et al. (2015)

Jung, K. U., et al. (2013)

Hu, M. G., et al. (2012)


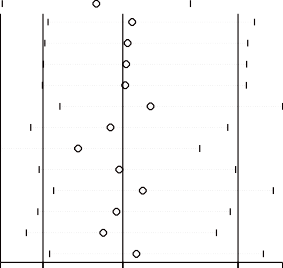
Huh, J. W., et al. (2011) Chen, K. Y., et al (2009)

Meta-analysis estimates, given named study is omitted Lower CI Limit Estimate Upper CI Limit

Ma, K., et al. (2018)

Ivanecz, A., et al. (2018) Xu, X., et al (2017)

Chen, Y. W., et al. (2017) Gorgun, E., et al. (2017) Ratti, F., et al. (2016) Tranchart et al. (2015) Lin, Q., et al. (2015)

Jung, K. U., et al. (2013)

Hu, M. G., et al. (2012)

Huh, J. W., et al. (2011) Chen, K. Y., et al (2009)

Meta-analysis estimates, given named study is omitted Lower CI Limit Estimate Upper CI Limit

Ma, K., et al. (2018)

Ivanecz, A., et al. (2018) Xu, X., et al (2017)

Chen, Y. W., et al. (2017) Gorgun, E., et al. (2017) Ratti, F., et al. (2016) Tranchart et al. (2015) Lin, Q., et al. (2015)

Jung, K. U., et al. (2013)

Hu, M. G., et al. (2012)

Huh, J. W., et al. (2011) Chen, K. Y., et al (2009)

Meta-analysis estimates, given named study is omitted Lower CI Limit Estimate Upper CI Limit

-206.44 -189.03

-113.31

-37.59 -20.70

1.39 7.80

36.57

65.35 74.14

0.35

0.46

0.66

0.96 1.07

#
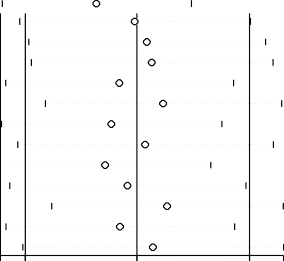
A B C

Ma, K., et al. (2018)

Meta-analysis estimates, given named study is omitted Lower CI Limit Estimate Upper CI Limit

Ma, K., et al. (2018)

Meta-analysis estimates, given named study is omitted Lower CI Limit Estimate Upper CI Limit

Ivanecz, A., et al. (2018)

Meta-analysis estimates, given named study is omitted Lower CI Limit Estimate Upper CI Limit

Ma, K., et al. (2018)

Meta-analysis estimates, given named study is omitted Lower CI Limit Estimate Upper CI Limit

Ivanecz, A., et al. (2018)

Xu, X., et al (2017)

Ivanecz, A., et al. (2018)

Xu, X., et al (2017)

Chen, Y. W., et al. (2017)

Gorgun, E., et al. (2017)

Xu, X., et al (2017)

Chen, Y. W., et al. (2017)

Tranchart et al. (2015)

Jung, K. U., et al. (2013)

Hu, M. G., et al. (2012)

Chen, Y. W., et al. (2017)

Tranchart et al. (2015)

Jung, K. U., et al. (2013)

Hu, M. G., et al. (2012)

Tranchart et al. (2015)

Lin, Q., et al. (2015)

Huh, J. W., et al. (2011)

Ratti, F., et al. (2016)

Jung, K. U., et al. (2013)

Huh, J. W., et al. (2011)

0.330.41


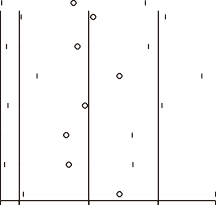
0.69

1.16 1.50

Huh, J. W., et al. (2011)

0.22


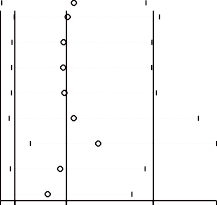

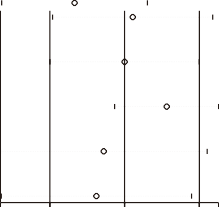
0.52

0.94

1.71 1.93

Chen, K. Y., et al (2009)

-4.34 -3.99

-2.70

-1.40 -0.33

Hu, M. G., et al. (2012)

-6.29

-5.06

-3.20

-1.34 -0.86

#
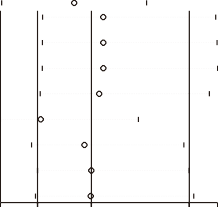
D E F G

Ma, K., et al. (2018)

Meta-analysis estimates, given named study is omitted Lower CI Limit Estimate Upper CI Limit

Ivanecz, A., et al. (2018)

Meta-analysis estimates, given named study is omitted Lower CI Limit Estimate Upper CI Limit

Jung, K. U., et al. (2013)

Meta-analysis estimates, given named study is omitted Lower CI Limit Estimate Upper CI Limit

Chen, Y. W., et al. (2017) Xu, X., et al (2017)

Tranchart et al. (2015) Chen, Y. W., et al. (2017)

Hu, M. G., et al. (2012)

Hu, M. G., et al. (2012)

Hu, M. G., et al. (2012)

Chen, K. Y., et al (2009) Huh, J. W., et al. (2011)

Ivanecz, A., et al. (2018)

0.170.23


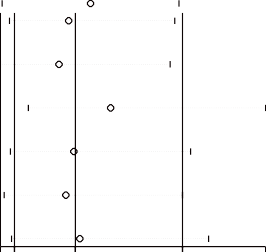
0.56

1.33 1.82

# H

Chen, K. Y., et al (2009)

0.44 0.53

0.94

1.65

# I

2.20

Chen, K. Y., et al (2009)

0.16 0.29


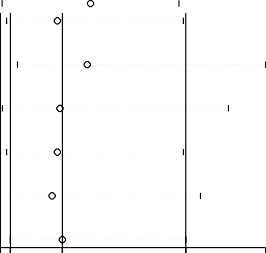
0.69

1.68 2.69

# J


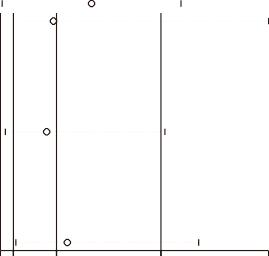
Figure S2. Sensitivity of all the outcomes. (A) Blood loss. (B) Operating time. (C) Postoperative complications. (D) Clavien grade<III

complications. (E) Clavien grade ≥III complications. (F) Hospital stay. (G) Postoperative stay. (H) One-year overall survival rate.

1. Three-year overall survival rate. (J) Five-year overall survival rate.

## Funnel plot with pseudo 95% conﬁdence limits


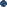

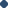

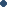

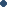

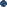

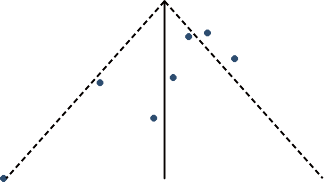

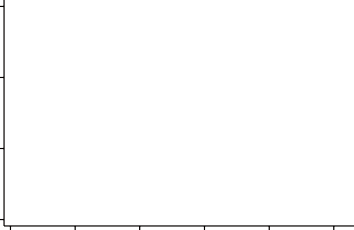


0

s.e. of logor

1.5 1 .5

Funnel plot with pseudo 95% conﬁdence limits


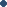

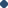

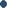

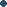

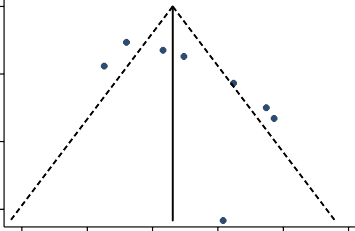


Standard error of WMD 100 50

Standard error of WMD 40 20

0

Funnel plot with pseudo 95% conﬁdence limits

-400 -300 -200 -100 0 100

150

60

0

WMD(Blood Loss)

# A

-100 -50 0 50 100 150

WMD(Operating Time)

# B

## -4 -2 0 2 4


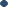

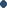

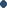

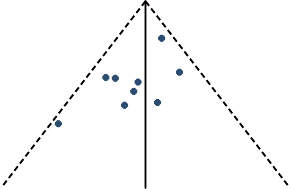

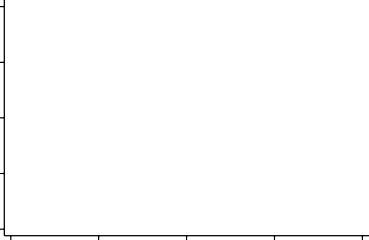


2

LogOR(Postoperative Complications)

C

Funnel plot with pseudo 95% conﬁdence limits


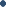

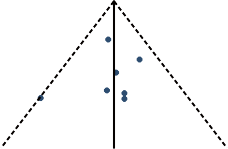

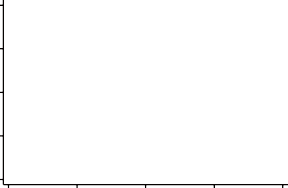


s.e. of logor

1 .5

0

-4 -2 0 2 4

2

1.5

LogOR(Clavien Grade<III Complications)

# D

Funnel plot with pseudo 95% conﬁdence limits

-4 -2 0 2 4


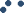

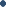

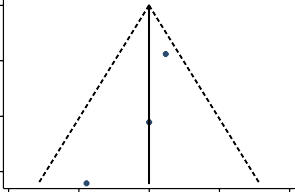


s.e. of logor

1 .5

1.5

0

Standard error of WMD 4 2



LogOR(Clavien Grade III Complications)

# E

Funnel plot with pseudo 95% conﬁdence limits


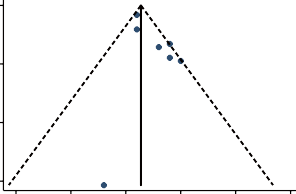
-15 -10 -5 0 5 10

6

0

Standard error of WMD 3 2 1

WMD(Hospital Stay)

# F

Funnel plot with pseudo 95% conﬁdence limits

-10 -5 0 5


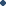

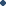

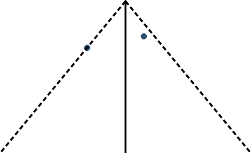

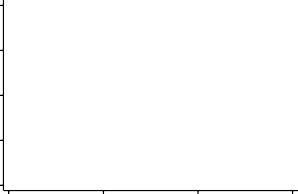


4

0

WMD(Postoperative Stay)

# G

##
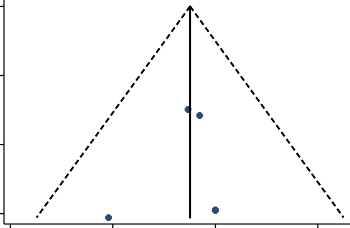
Funnel plot with pseudo 95% conﬁdence limits

s.e. of logor

1 .5

0

-4 -2 0 2

1.5

LogOR(One-year Overall Survival Rate)

## Funnel plot with pseudo 95% conﬁdence limits

-4 -2 0 2


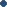

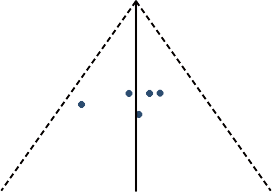

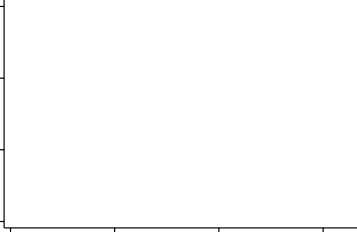


s.e. of logor

1 .5

1.5

0

LogOR(Three-year Overall Survival Rate)

## Funnel plot with pseudo 95% conﬁdence limits

-4 -2 0 2 4


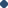

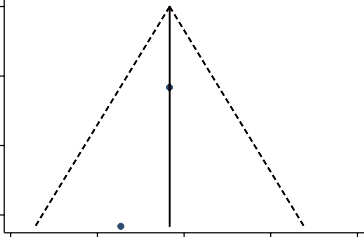


s.e. of logor

1 .5

1.5

0

LogOR(Five-year Overall Survival Rate)

# H I J

Quantitative Assessment for Publication Bias

## Postoperative

complications

| Number of studies | 12 | 12 | 12 | 7 | 5 | 6 | 6 | 3 |
| --- | --- | --- | --- | --- | --- | --- | --- | --- |
| P value for Harbord tests | 0.524 | - | - | - | - | 0.929 | 0.560 | 0.483 |
| P value for Peters tests | 0.155 | - | - | - | - | 0.012 | 0.584 | - |
| P value for Egger tests | - | 0.774 | 0.030 | 0.086 | 0.575 | - | - | - |

K

Blood loss Operating time Hospital stay Postoperative stay One-year overall

survival rate

Three-year overall survival rate

Five-year overall survival rate

Figure S3. Publication bias of all the outcomes. (A)-(J) Funnel plot of Blood loss, Operating time, Postoperative complications, Clavien grade<III complications, Clavien grade ≥III complications, Hospital stay, Postoperative stay, One-year overall survival rate, Three-year overall survival rate, Five-year overall survival rate. (K) Quantitative assessment for publication bias.
