# Supplementary material for: Laparoscopic procedure is associated with lower morbidity for simultaneous resection of colorectal cancer and liver metastases: an updated meta-analysis
Source: World J Surg Oncol. 2020 Sep 21;18:251. doi: 10.1186/s12957-020-02018-z (PMC7507629; doi:10.1186/s12957-020-02018-z)
Supplement: Supplementary file 1 — Additional file 1: Figure S1. Forest plot of meta-analysis. (A) Blood loss. (B) Operating time. (C) Subgroup analysis of postoperative complications. (D) Clavien grade<III complications. (E) Clavien grade ≥III complications. (F) Hospital stay. (G) Postoperative stay. (H) One-year overall survival rate. (I) Three-year overall survival rate. (J) Five-year overall survival rate. Figure S2. Sensitivity of all the outcomes. (A) Blood loss. (B) Operating time. (C) Postoperative complications. (D) Clavien grade<III complications. (E) Clavien grade ≥III complications. (F) Hospital stay. (G) Postoperative stay. (H) One-year overall survival rate. (I) Three-year overall survival rate. (J) Five-year overall survival rate. Figure S3. Publication bias of all the outcomes. (A)-(J) Funnel plot of Blood loss, Operating time, Postoperative complications, Clavien grade<III complications, Clavien grade ≥III complications, Hospital stay, Postoperative stay, One-year overall survival rate, Three-year overall survival rate, Five-year overall survival rate. (K) Quantitative assessment for publication bias. Table S1. Search Strategy for Each Database. Table S2. Quality Assessment of Included Studies. Table S3. Excluded Articles and Reasons for Exclusion. [file 12957_2020_2018_MOESM1_ESM.zip › Additional Tables.docx]

**Table S1. Search Strategy**

| **Database** | **Search strategy** |
| --- | --- |
| PubMed | #1”Colorectal Neoplasms”[Mesh] |
|  | #2 (“colorectal neoplasm”[Title/Abstract] OR “colorectal cancer”[Title/Abstract] OR “colon cancer” [Title/Abstract] OR “colorectal tumor*” [Title/Abstract] OR “colon carcinoma” [Title/Abstract] OR “rectal cancer” [Title/Abstract] OR “rectal carcinoma” [Title/Abstract]) |
|  | #3 (“colorectal cancer liver metastases” [Title/Abstract] OR “colorectal liver metastases” [Title/Abstract] OR “resectable liver metastases from colorectal cancer” [Title/Abstract] OR “metastatic hepatic cancer with colorectal carcinoma” [Title/Abstract] OR “hepatic metastasis of colonic carcinoma” [Title/Abstract]) |
|  | #4 #1 OR #2 OR #3 |
|  | #5 ("neoplasm"[Title/Abstract] AND "metastasis" [Title/Abstract]) |
|  | #6 “Neoplasm Metastasis” [Mesh] |
|  | #7 (“neoplasm metastasis” [Title/Abstract] OR metasta*[Title/Abstract]) |
|  | #8 #5 OR #6 OR #7 |
|  | #9 (liver[Title/Abstract] OR hepatic[Title/Abstract]) |
|  | #10 Liver[Mesh] |
|  | #11 #9 OR #10 |
|  | #12 (synchronous[Title/Abstract] OR simultaneous[Title/Abstract] OR combined[Title/Abstract] OR concurrent[Title/Abstract]) |
|  | #13 (robot* [Title/Abstract] OR "minimally invasive"[Title/Abstract] ) |
|  | #14 (laparoscopy[Title/Abstract] OR laparoscopic[Title/Abstract]) |
|  | #15 Laparoscopy[Mesh] |
|  | #16 #13 OR #14 OR #15 |
|  | #17 (hepatectomy[Title/Abstract] OR “liver resection”[Title/Abstract] OR “hepatic resection”[Title/Abstract]) |
|  | #18 (laparotomy[Title/Abstract] OR open[Title/Abstract]) |
|  | #19 Laparotomy[Mesh] |
|  | #20 #17 OR #18 OR #19 |
|  | #21 #4 AND #8 AND #11 AND #12 AND #16 AND#20 |

| **Database** | **Search strategy** |
| --- | --- |
| WOS | #1. TOPIC: ("colorectal neoplasm") OR TOPIC: ("colorectal cancer") OR TOPIC: ("colon cancer") OR TOPIC: ("colorectal tumor*") OR TOPIC: ("colon carcinoma") OR TOPIC: ("rectal cancer") OR TOPIC: ("rectal carcinoma") |
|  | #2. TOPIC: ("colorectal cancer liver metastases") *OR* TOPIC:  ("colorectal liver metastases") *OR* TOPIC: ("resectable liver metastases from colorectal cancer") *OR* TOPIC: ("metastatic hepatic cancer with colorectal carcinoma") *OR* TOPIC: ("hepatic metastasis of colonic carcinoma") |
|  | #3. #2 OR #1 |
|  | #4. TOPIC: (neoplasm) *AND* TOPIC: (metastasis) |
|  | #5. TOPIC: ("neoplasm metastasis") *OR* TOPIC: (metasta*) |
|  | #6. #5 OR #4 |
|  | #7. TOPIC: (liver) *OR* TOPIC: (hepatic) |
|  | #8. TOPIC: (synchronous) *OR* TOPIC: (simultaneous) *OR* TOPIC:   (combined) *OR* TOPIC: (concurrent) |
|  | #9. TOPIC: (robot*) *OR* TOPIC: ("minimally invasive") |
|  | #10. TOPIC: (laparoscopy) *OR* TOPIC: (laparoscopic) |
|  | #11. #10 OR #9 |
|  | #12. TOPIC: (hepatectomy) *OR* TOPIC: ("liver resection") *OR* TOPIC:   ("hepatic resection") |
|  | #13. TOPIC: (Laparotomy) *OR* TOPIC: (open) |
|  | #14. #13 OR #12 |
|  | #15. #14 AND #11 AND #8 AND #7 AND #6 AND #3 |

| **Database** | **Search strategy** |
| --- | --- |
| Embase | #1. 'colorectal tumor'/exp |
|  | #2. 'colorectal neoplasm':ab,ti OR 'colorectal  cancer':ab,ti OR 'colon cancer':ab,ti OR  'colorectal tumor*':ab,ti OR 'colon  carcinoma':ab,ti OR 'rectal cancer':ab,ti OR  'rectal carcinoma':ab,ti |
|  | #3. 'colorectal cancer liver metastases':ab,ti OR  'colorectal liver metastases':ab,ti OR  'resectable liver metastases from colorectal  cancer':ab,ti OR 'metastatic hepatic cancer with  colorectal carcinoma':ab,ti OR 'hepatic |
|  | #4. #1 OR #2 OR #3 |
|  | #5. 'neoplasm':ab,ti AND 'metastasis':ab,ti |
|  | #6. 'metastasis'/exp |
|  | #7. 'neoplasm metastasis':ab,ti OR 'metasta*':ab,ti |
|  | #8. #5 OR #6 OR #7 |
|  | #9. 'liver':ab,ti OR 'hepatic':ab,ti |
|  | #10. 'liver'/exp |
|  | #11. #9 OR #10 |
|  | #12. 'synchronous':ab,ti OR 'simultaneous':ab,ti OR  'combined':ab,ti OR 'concurrent':ab,ti |
|  | #13. 'robot*':ab,ti OR 'minimally invasive':ab,ti OR  'laparoscopy':ab,ti OR 'laparoscopic':ab,ti |
|  | #14. 'laparoscopy':ab,ti OR 'laparoscopic':ab,ti |
|  | #15. 'laparoscopy'/exp |
|  | #16. #13 OR #14 OR #15 |
|  | #17. 'hepatectomy':ab,ti OR 'resection':ab,ti |
|  | #18. 'laparotomy':ab,ti OR 'open':ab,ti |
|  | #19. 'laparotomy'/exp |
|  | #20. 'liver resection'/exp |
|  | #21. #17 OR #18 OR #19 OR #20 |
|  | #22. #4 AND #8 AND #11 AND #12 AND #16 AND #21 |

| **Database** | **Search strategy** |
| --- | --- |
| Cochrane | #1 MeSH descriptor: [Colorectal Neoplasms] explode all trees |
| Library | #2 (colorectal neoplasm):ti,ab,kw OR ("colorectal cancer"):ti,ab,kw OR ("colon cancer"):ti,ab,kw OR (colorectal tumor):ti,ab,kw OR (colon carcinoma):ti,ab,kw (Word variations have been searched) |
|  | #3 ("rectal cancer"):ti,ab,kw OR (rectal carcinoma):ti,ab,kw (Word variations have been searched) |
|  | #4 (colorectal cancer liver metastases):ti,ab,kw OR (colorectal liver metastases):ti,ab,kw OR (resectable liver metastases from colorectal cancer):ti,ab,kw OR (metastatic hepatic cancer with colorectal carcinoma):ti,ab,kw OR (hepatic metastasis of colonic carcinoma):ti,ab,kw (Word variations have been searched) |
|  | #5 #1 OR #2 OR #3 OR #4 |
|  | #6 ("neoplasm"):ti,ab,kw AND ("metastasis"):ti,ab,kw (Word variations have been searched) |
|  | #7 MeSH descriptor: [Neoplasm Metastasis] explode all trees |
|  | #8 (neoplasm metastasis):ti,ab,kw OR (metasta*):ti,ab,kw (Word variations have been searched) |
|  | #9 #6 OR #7 OR #8 |
|  | #10 ("liver"):ti,ab,kw OR ("hepatic"):ti,ab,kw (Word variations have been searched) |
|  | #11 MeSH descriptor: [Liver] explode all trees |
|  | #12 #10 OR #11 |
|  | #13 ("synchronous"):ti,ab,kw OR ("simultaneous"):ti,ab,kw OR (combined):ti,ab,kw OR ("concurrent"):ti,ab,kw (Word variations have been searched) |
|  | #14 (robot*):ti,ab,kw OR (minimally invasive):ti,ab,kw (Word variations have been searched) |
|  | #15 ("laparoscopy"):ti,ab,kw OR ("laparoscopic"):ti,ab,kw (Word variations have been searched) |
|  | #16 MeSH descriptor: [Laparoscopy] explode all trees |
|  | #17 #14 OR #15 OR #16 |
|  | #18 ("hepatectomy"):ti,ab,kw OR (liver resection):ti,ab,kw OR (hepatic resection):ti,ab,kw (Word variations have been searched) |
|  | #19 ("laparotomy"):ti,ab,kw OR ("open"):ti,ab,kw (Word variations have been searched) |
|  | #20 MeSH descriptor: [Laparotomy] explode all trees |
|  | #21 #18 OR #19 OR #20 |
|  | #22 #5 AND #9 AND #12 AND #13 AND #17 AND #21 |

**Table S2. Quality Assessment of Included Studies**

| **Study** | **Selection** | **Comparability** | **Outcome** | **Quality Score** |
| --- | --- | --- | --- | --- |
| Ma et al[1], 2018 | 4 | 1 | 3 | 8 |
| Ivanecz et al[2], 2018 | 4 | 1 | 3 | 8 |
| Xu et al[3], 2017 | 4 | 1 | 3 | 8 |
| Chen et al[4], 2018 | 3 | 1 | 2 | 6 |
| Gorgun et al[5], 2017 | 3 | 1 | 2 | 6 |
| Ratti et al[6], 2016 | 4 | 1 | 3 | 8 |
| Tranchart et al[7], 2016 | 4 | 1 | 3 | 8 |
| Lin et al[8], 2015 | 4 | 1 | 3 | 8 |
| Jung et al[9], 2013 | 3 | 1 | 2 | 6 |
| Hu et al[10], 2012 | 4 | 1 | 3 | 8 |
| Huh et al[11], 2011 | 4 | 1 | 3 | 8 |
| Chen et al[12], 2011 | 4 | 1 | 3 | 8 |

**Reference**

1. Ma K, Wang XY, Chen JH: **[Laparoscopic versus open surgery for simultaneous resection of synchronous colorectal liver metastases].** *Zhonghua Wai Ke Za Zhi* 2018, **56:**516-521.

2. Ivanecz A, Krebs B, Stozer A, Jagric T, Plahuta I, Potrc S: **Simultaneous pure laparoscopic resection of primary colorectal cancer and synchronous liver metastases: a single institution experience with propensity score matching analysis.** *Radiology and Oncology* 2017, **52:**42-53.

3. Xu X, Guo Y, Chen G, Li C, Wang H, Dong G: **Laparoscopic resections of colorectal cancer and synchronous liver metastases: a case controlled study.** *Minim Invasive Ther Allied Technol* 2018, **27:**209-216.

4. Chen YW, Huang MT, Chang TC: **Long term outcomes of simultaneous laparoscopic versus open resection for colorectal cancer with synchronous liver metastases.** *Asian J Surg* 2019, **42:**217-223.

5. Gorgun E, Yazici P, Onder A, Benlice C, Yigitbas H, Kahramangil B, Tasci Y, Aksoy E, Aucejo F, Quintini C, et al: **Laparoscopic versus open 1-stage resection of synchronous liver metastases and primary colorectal cancer.** *Gland Surgery* 2017, **6:**324-329.

6. Ratti F, Catena M, Di Palo S, Staudacher C, Aldrighetti L: **Impact of totally laparoscopic combined management of colorectal cancer with synchronous hepatic metastases on severity of complications: a propensity-score-based analysis.** *Surg Endosc* 2016, **30:**4934-4945.

7. Tranchart H, Fuks D, Vigano L, Ferretti S, Paye F, Wakabayashi G, Ferrero A, Gayet B, Dagher I: **Laparoscopic simultaneous resection of colorectal primary tumor and liver metastases: a propensity score matching analysis.** *Surg Endosc* 2016, **30:**1853-1862.

8. Lin Q, Ye Q, Zhu D, Wei Y, Ren L, Zheng P, Xu P, Ye L, Lv M, Fan J, Xu J: **Comparison of minimally invasive and open colorectal resections for patients undergoing simultaneous R0 resection for liver metastases: a propensity score analysis.** *Int J Colorectal Dis* 2015, **30:**385-395.

9. Jung KU, Kim HC, Cho YB, Kwon CHD, Yun SH, Heo JS, Lee WY, Chun HK: **Outcomes of Simultaneous Laparoscopic Colorectal and Hepatic Resection for Patients with Colorectal Cancers: A Comparative Study.** *Journal of Laparoendoscopic & Advanced Surgical Techniques* 2014, **24:**229-235.

10. Hu MG, Ou-Yang CG, Zhao GD, Xu DB, Liu R: **Outcomes of Open Versus Laparoscopic Procedure for Synchronous Radical Resection of Liver Metastatic Colorectal Cancer: A Comparative Study.** *Surgical Laparoscopy Endoscopy & Percutaneous Techniques* 2012, **22:**364-369.

11. Huh JW, Koh YS, Kim HR, Cho CK, Kim YJ: **Comparison of laparoscopic and open colorectal resections for patients undergoing simultaneous R0 resection for liver metastases.** *Surg Endosc* 2011, **25:**193-198.

12. Chen KY, Xiang GA, Wang HN, Xiao FL: **Simultaneous laparoscopic excision for rectal carcinoma and synchronous hepatic metastasis.** *Chin Med J (Engl)* 2011, **124:**2990-2992.

**Table S3. Excluded Articles and Reasons for Exclusion**

| **Excluded Articles** | **Reasons for exclusion** |
| --- | --- |
| Yang, T.X 2013[1] | This article is a meta-analysis. |
| Guo, Y.L 2018[2] | This article is a meta-analysis. |
| Xie, S.M 2017[3] | This article is a meta-analysis. |
| Wei, M 2014[4] | This article is a meta-analysis. |
| Ciria, R 2016[5] | This article is a meta-analysis. |
| Ito, T 2016[6] | This article is a case-report. |
| Fornseca Buitrago, C.L 2017[7] | This article is a case-report. |
| Aral, K 2017[8] | This article is a case-report. |
| Shinohara, S 2018[9] | This article is a case-report. |
| Xiao, N 2017[10] | This article is a case-report. |
| Jin, H 2016[11] | This article is a case-report. |
| Berti, S 2017[12] | This article is a case-report. |
| Saeed, M 2018[13] | This article is a case-report. |
| Van Bael, K 2015[14] | This article is a case-report. |
| Kodai, S 2017[15] | This article is a conference summary. |
| Ratti, F 2016[16] | This article is a conference summary. |
| Ratti, F 2014[17] | This article is a conference summary. |
| Ratti, F 2011[18] | This article is a conference summary. |
| Slavchev, M 2015[19] | This article is a conference summary. |
| Xu, J 2014[20] | This article is a conference summary. |
| Eveno, C 2016[21] | This article is a conference summary. |
| Shin, J.K 2016[22] | This article is a conference summary. |
| Ratti, F 2010[23] | This article is a conference summary. |
| Cipriani, F 2016[24] | This article is a conference summary. |
| Cipriani, F 2017[25] | This article is a conference summary. |
| Gorgun, E 2015[26] | This article is a conference summary. |
| Taesomabt, W 2018[27] | This article is a conference summary. |
| Cipriani, F 2016[28] | Some of patients have metachronous instead of synchronous metastases. |
| Shim, J.R 2018[29] | Some of patients have metachronous instead of synchronous metastases. |
| Fretland, AA 2015[30] | The included patients do not undergo simultaneous resection. |
| Cannon, R.M 2012[31] | The included patients do not undergo simultaneous resection. |
| Untereiner, X 2016[32] | Some of patients have metachronous instead of synchronous metastases. |
| Takasu. C 2014[33] | The number of included patients is lower than 20. |

**Reference**

1. Yang TX, Billah B, Morris DL, Chua TC: **Palliative resection of the primary tumour in patients with Stage IV colorectal cancer: systematic review and meta-analysis of the early outcome after laparoscopic and open colectomy.** *Colorectal Disease* 2013, **15:**e407-e419.

2. Guo YL, Gao YH, Chen GJ, Li C, Dong GL: **Minimally Invasive versus Open Simultaneous Resections of Colorectal Cancer and Synchronous Liver Metastases: A Meta-Analysis.** *American Surgeon* 2018, **84:**192-200.

3. Xie SM, Xiong JJ, Liu XT, Chen HY, Iglesia-Garcia D, Altaf K, Bharucha S, Huang W, Nunes QM, Szatmary P, Liu XB: **Laparoscopic Versus Open Liver Resection for Colorectal Liver Metastases: A Comprehensive Systematic Review and Meta-analysis.** *Scientific Reports* 2017, **7**.

4. Wei M, He Y, Wang J, Chen N, Zhou Z, Wang Z: **Laparoscopic versus open hepatectomy with or without synchronous colectomy for colorectal liver metastasis: a meta-analysis.** *PLoS One* 2014, **9:**e87461.

5. Ciria R, Cherqui D, Geller DA, Briceno J, Wakabayashi G: **Comparative Short-term Benefits of Laparoscopic Liver Resection: 9000 Cases and Climbing.** *Annals of Surgery* 2016, **263:**761-777.

6. Ito T, Kushida T, Sakurada M, Maekawa H, Orita H, Senuma K, Mizuguchi K, Sato K: **Two cases of laparoscopic-assisted simultaneous resection of colorectal cancer and synchronous liver metastases in elderly patients.** *Surgical Endoscopy and Other Interventional Techniques* 2016, **30:**S341.

7. Fonseca Buitrago CL, Sánchez Martinez LM, Daza FP, Ruiz CA, Cárdenas AM, Vega J, Cabrera M, Arenas NJ: **Synchronous colorectal and renal cell carcinoma: A case report.** *Urologia Colombiana* 2017, **26:**159-163.

8. Arai K, Kido M, Kinoshita H, Fukumoto T, Tanaka M, Kuramitsu K, Matsumoto T, Toyama H, Asari S, Goto T, et al: **Simultaneous laparoscopic resection of colorectal cancer and synchronous metastatic liver tumor: A case report.** *Surgical Endoscopy and Other Interventional Techniques* 2017, **31:**S390.

9. Shinohara S, Hyodo M, Ota G, Hayashi H, Inoue Y, Sato H, Tsukahara M, Okada M, Lefor AK, Yasuda Y: **[Primary Small Bowel Tumor with Simultaneous Lung Metastases from Rectal Cancer - A Case Report].** *Gan To Kagaku Ryoho* 2018, **45:**1377-1379.

10. Xiao N, Yu K, Yu S, Wu J, Wang J, Shan S, Zheng S, Wang L, Wang J, Peng S: **The paradigm of tumor shrinkage and rapid liver remnant hypertrophy for conversion of initially unresectable colorectal liver metastasis: a case report and literature review.** *World J Surg Oncol* 2017, **15:**148.

11. Jin H, Morohashi H, Sato K, Umetsu S, Yoshida T, Wakasa Y, Ichinohe D, Ogasawara H, Hasebe T, Miura T, et al: **Laparoscopic Lateral Lymph Node Dissection for Rectal Cancer with Lateral Lymph Node Metastasis - A Case Report.** *Gan to kagaku ryoho Cancer & chemotherapy* 2016, **43:**2292-2294.

12. Berti S, Francone E, Gennai A, Muzio E, Sagnelli C, Falco E: **Incisionless combined total laparoscopic approach for hepatic and colorectal resection (Tatme): Case report.** *Techniques in Coloproctology* 2017, **21:**80.

13. Saeed M, Tat C, Kaminski J, Estrada J: **Colorectal cancer and acromegaly: A case report and review of the current guidelines.** *Surgical Endoscopy and Other Interventional Techniques* 2018, **32:**S211.

14. Van Bael K, Jansen Y, Seremet T, Engels B, Delvaux G, Neyns B: **A case report of long-term survival following hepatic arterial infusion of l-folinic acid modulated 5-fluorouracil combined with intravenous irinotecan and cetuximab followed by hepatectomy in a patient with initially unresectable colorectal liver metastases.** *Case Reports in Oncological Medicine* 2015, **2015**.

15. Kodai S, Kanazawa A, Nozawa A, Murata A, Shimizu S, Deguchi S, Tashima T, Tauchi J, Miura K, Urata Y: **Usefulness of the laparoscopic liver resection for colorectal liver metastases.** *Journal of Hepato-Biliary-Pancreatic Sciences* 2017, **24:**A284.

16. Ratti F, Catena M, Di Palo S, Staudacher C, Aldrighetti L: **Synchronous surgery for colorectal cancer and liver metastases: Laparoscopic colorectal resection reduces postoperative morbidity index. A case matched analysis using propensity scores.** *HPB* 2016, **18:**e729.

17. Ratti F, Aldrighetti L, Catena M, Di Palo S, Staudacher C, Ferla G: **Synchronous surgery for colorectal cancer and liver metastases: Laparoscopic colorectal resection improves short term outcome. A comparative study.** *HPB* 2014, **16:**123.

18. Ratti F, Aldrighetti L, Di Palo S, Orsenigo E, Catena M, Guzzetti E, Ghirardelli L, Staudacher C, Ferla G: **Synchronous colorectal liver metastases: Laparoscopic or open approach for colorectal surgery?** *HPB* 2011, **13:**90-91.

19. Slavchev M, Atanasov B, Belev N, Krstev P: **Simultaneous laparoscopic procedures for colorectal cancer with synchronous liver metastases.** *Colorectal Disease* 2015, **17:**84.

20. Xu J, Lin Q, Zhu D, Ye Q: **Short-and long-term outcomes in simultaneous resection of colorectal cancer and liver metastasis with minimally invasive or open surgery.** *Journal of Clinical Oncology* 2014, **32**.

21. Eveno C, Cailliez V, Lopezben S, Mirza DF, Kaiser G, Lapointe R, Mentha G, Pardo F, Adam R, Cherqui D: **Role of laparoscopic approach in colorectal liver metastasis. An international multi-center data analysis using liver met survey.** *HPB* 2016, **18:**e61-e62.

22. Shin JK, Kim HC, Lee WY, Yun SH, Cho YB, Huh JW, Park YA: **Laparoscopic versus open liver resection for metastatic colorectal cancer: A comparative study.** *Colorectal Disease* 2016, **18:**103.

23. Ratti F, Aldrighetti L, Di Palo S, Orsenigo E, Catena M, Guzzetti E, Ghirardelli L, Staudacher C, Ferla G: **Laparoscopic and open approach for synchronous colorectal liver metastases.** *European Surgical Research* 2010, **45:**220.

24. Cipriani F, Rawashdeh M, Stanto L, Armstrong T, Takhar A, Pearce N, Primrose J, Abu Hilal M: **Impact of the laparoscopic approach on short-and longterm outcomes in patients with colorectal liver metastases. A propensity score analysis on a single centre experience.** *HPB* 2016, **18:**e76.

25. Cipriani F, Rawashdeh M, Stanton L, Armstrong T, Takhar A, Pearce NW, Primrose JN, Hilal MA, Barbaro S: **Impact of the laparoscopic approach for colorectal liver metastases on short- and long-term outcomes. A propensity score analysis.** *Surgical Endoscopy and Other Interventional Techniques* 2017, **31:**S444.

26. Gorgun E, Yazici P, Onder A, Sound S, Okoh AK, Tasci Y, Aksoy E, Aucejo FN, Quintini C, Miller CM, et al: **Concomitant surgical management of synchronous colorectal cancer and liver metastases: A comparison of open and laparoscopic approaches.** *Gastroenterology* 2015, **148:**S1126.

27. Taesombat W, Nonthasoot B, Vorasittha A, Sutherasan M, Nivatvongs S, Sirichindakul B: **Benefits of simultaneous totally laparoscopic colorectal surgery and liver resection for colorectal cancer with synchronous liver metastases.** *Surgical Endoscopy and Other Interventional Techniques* 2018, **32:**S277.

28. Cipriani F, Rawashdeh M, Stanton L, Armstrong T, Takhar A, Pearce NW, Primrose J, Abu Hilal M: **Propensity score-based analysis of outcomes of laparoscopic versus open liver resection for colorectal metastases.** *Br J Surg* 2016, **103:**1504-1512.

29. Shim JR, Lee SD, Park HM, Lee EC, Park B, Han SS, Kim SH, Park SJ: **Outcomes of liver resection in patients with colorectal liver metastases by laparoscopic or open surgery.** *Ann Hepatobiliary Pancreat Surg* 2018, **22:**223-230.

30. Fretland Å, Kazaryan AM, Bjørnbeth BA, Flatmark K, Andersen MH, Tønnessen TI, Bjørnelv GM, Fagerland MW, Kristiansen R, Øyri K, et al.: **Open versus laparoscopic liver resection for colorectal liver metastases (the Oslo-CoMet Study): study protocol for a randomized controlled trial.** *Trials* 2015, **16:**73.

31. Cannon RM, Scoggins CR, Callender GG, McMasters KM, Martin RC, 2nd: **Laparoscopic versus open resection of hepatic colorectal metastases.** *Surgery* 2012, **152:**567-573; discussion 573-564.

32. Untereiner X, Cagniet A, Memeo R, Tzedakis S, Piardi T, Severac F, Mutter D, Kianmanesh R, Marescaux J, Sommacale D, Pessaux P: **Laparoscopic hepatectomy versus open hepatectomy for colorectal cancer liver metastases: comparative study with propensity score matching.** *Hepatobiliary Surg Nutr* 2016, **5:**290-299.

33. Takasu C, Shimada M, Sato H, Miyatani T, Imura S, Morine Y, Ikemoto T, Kanamoto M, Kurita N, Eto S, Utsunomiya T: **Benefits of simultaneous laparoscopic resection of primary colorectal cancer and liver metastases.** *Asian J Endosc Surg* 2014, **7:**31-37.
